# Tenosynovial giant cell tumour of the finger: a case report

**DOI:** 10.11604/pamj.2023.45.49.37714

**Published:** 2023-05-19

**Authors:** Ankit Jaiswal, Ratnakar Ambade

**Affiliations:** 1 Department of Orthopaedics, Jawaharlal Nehru Medical College, Datta Meghe Institute of Higher Education and Research (Deemed to be University), Wardha, Maharashtra, India

**Keywords:** Giant cell tumour, bone tumour, resection, orthoplasty, case report

## Abstract

Giant cell tumour most commonly occuring in epiphysis of the long bone, present and with pain, tenderness and swelling. It is a solitary lesion with restricted movement and tenderness over the lesion. The tendon sheath is where tenosynovial giant cell tumours typically develop. Because of its remarkably peculiar position, we present a case of giant cell tumour (GCT) tenosynovial of bone in the middle phalaynx in a 33-year-old female with complaints of swelling, pain in ring finger of left hand since 2 months which is rarely seen. After clinical, radiological, pathological investigations tenosynovial giant cell tumour was diagnosed. Following fine needle aspiration cytology, histopathology was utilized to confirm the tumour's diagnosis which was later treated as resection of excision of the tumour with allo/autograft reconstruction. Our case report showed no evidence of recurrence in 2 years of follow-up. Hence our case report proves that early and complete resection of the tumour shows evidence of regain of complete range of motion and decrease recurrence rate.

## Introduction

Extremely seldomly, a bone giant-cell tumour (GCT) can develop in the phalanx of a finger. Giant cell tumour of the tendon sheath, also known as tenosynovial giant cell tumour (GCT-TS), is a single, hard, benign, nodular soft-tissue tumour that develops from the synovial lining of a tendon. The cause is not known. It is an uncommon soft-tissue tumour that often affects adults between the ages of 30 and 50. It has an overall incidence of one in 50,000 people. When it comes to imaging techniques for surgical planning, magnetic resonance imaging is frequently done [1]. The most recent (2013) World Health Organization classification uses the term “tenosynovial giant cell tumour” [2]. The epiphysis of a long bone is frequently the site of a giant cell tumour (GCT), a rare type of benign osseous tumour that develops after skeletal development. The most typical signs of a giant cell tumour are as follows. Some signs might be: a definite mass, associated with fractured bones, fluid accumulation in the joint closest to the injured bone, proximal joint restricted, pain and swelling with discomfort at the closest joint. The clinical findings of this rare tumour are most commonly located within epiphysis of long bone but extend into metaphysis. It is a solitary lesion with restricted movement and tenderness over the lesion. It is categorized as a benign tumour that is locally spreading. Only 2% of all GCT instances that have been recorded have included the hand, and phalangeal bone involvement as the predominant symptom is exceedingly rare. The behaviour of GCTs at phalanxes has shown to be substantially different from that of GCTs in more usual locations, such as the distal femur, with greater rates of recurrence [2].

## Patient and observation

**Patient and observation:** a 33-year-old woman complained of soreness and swelling in the left ring finger's middle phalanx over the previous two months. No prior injuries or signs of a related constitutional condition existed. With only minor pain, the swelling had been progressively growing in size. The discomfort was mild, constant, without a daily cycle, localized to the lesion site, and non-radiating. Fever, appetite loss, additional joint involvement, or swelling over any other body part are not past occurrences. No significant past, medical, family, psycho-social or genetic history.

**Figure 1:**
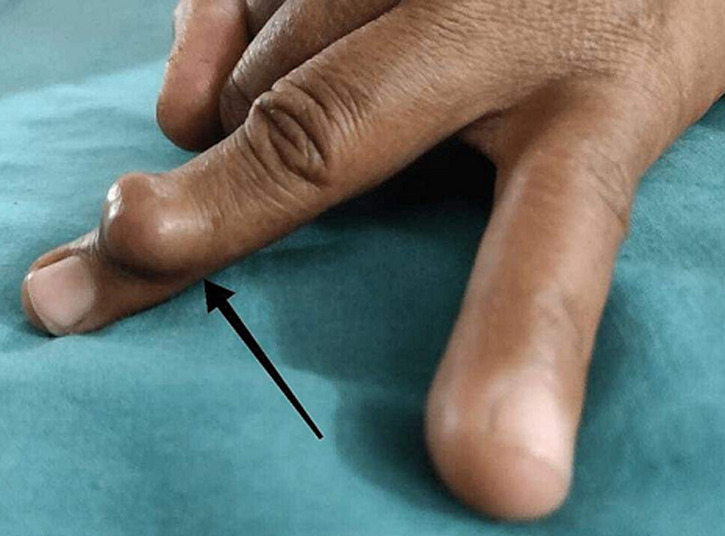
swelling on the left ring finger from the dorsal, and lateral aspects

**Clinical findings:** upon examination, it was discovered that the left ring finger's middle phalanx had a little (2x2x1) localized edema and dull pain with no aggravating or relieving factors. On the left side of the ring finger, the swelling was globular in shape, grape like, bony hard in consistency with localized tenderness, restricted to the distal interphalangeal joint and moved along with the tendon of the muscle but was fixed when the muscle was made taught against the resistance. Normal skin underneath was stiff and showed no symptoms of inflammation, localized temperature increase, or nasal discharge (Figure 1). The skin over the lesion is pinchable. The ring finger's distal interphalangeal joint (DIP joint) was uncomfortable and limited in mobility. When the distal interphalangeal joint and proximal interphalangeal joint moves, swelling also was mobile. No concomitant lymphadenopathy existed. The nail bed's capillary refilling was good. Allen's test revealed the patent status of both digital vessels [3]. No sensory deficit was present.

**Diagnostic assessment:** the middle end of the metaphysis and the middle phalanx's diaphysis were both affected by the tumour, which was visible on X-ray of hand antero-posterior and oblique view as an expansile, osteolytic-lobulated soap bubble. An incision was made to treat the patient, and the swelling on the left ring finger was drained. There was no periosteal response or rupture of the DIP joint's articular surface. The tumour's additional foci were not visible. Serum calcium, other electrolytes and alkaline phosphatase levels were all within normal limits according to laboratory results.

**Cytologic findings:** a 22-gauge needle was used to do fine-needle aspiration on the lesion. Giemsa's stain was used to stain the produced smears [4]. Giant cell tumour cells were seen on smears. Giant multinucleated cells (osteoclasts): these cells possessed multiple bland nuclear chromatids that were ovoid or spherical, much like mononuclear cells. In most microscopic fields, these cells were affixed to the edges of mononuclear cell clusters.

**Figure 2:**
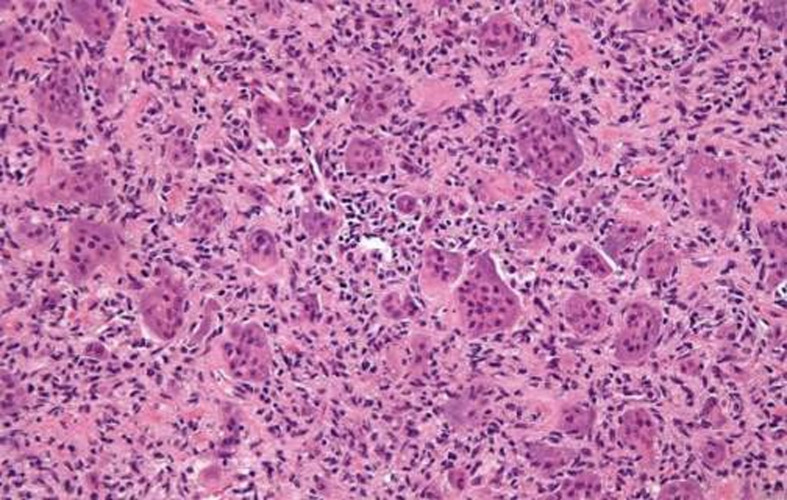
histopathology section showing uniform admixture of multinucleated giant cells and stromal cells

**Histological findings:** sections underwent histological analysis, which revealed an admixture of osteoclastic large cells grouped in a predictable pattern (Figure 2). No signs of necrosis, atypia, or mitosis were seen. In the histological sections, there were not many places where new bone was growing, though. In agreement with the cytological diagnosis, giant-cell tenosynovitis was identified.

**Diagnosis:** Enneking *et al*. [4] proposed a clinico- radiographical system for grading of GCT tumours according to which this case had a grade 1 tenosynovial GCT benign, biologically static, well-defined margin and indolent soft tissue mass.

**Figure 3:**
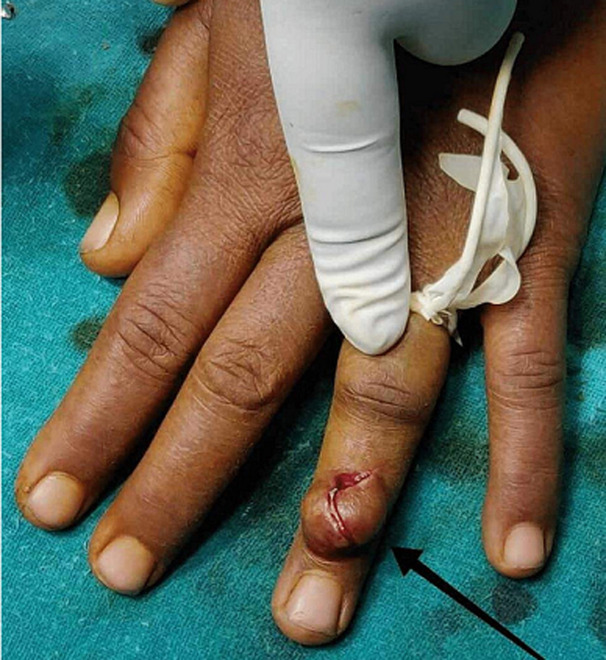
intraoperative image of incision

**Figure 4:**
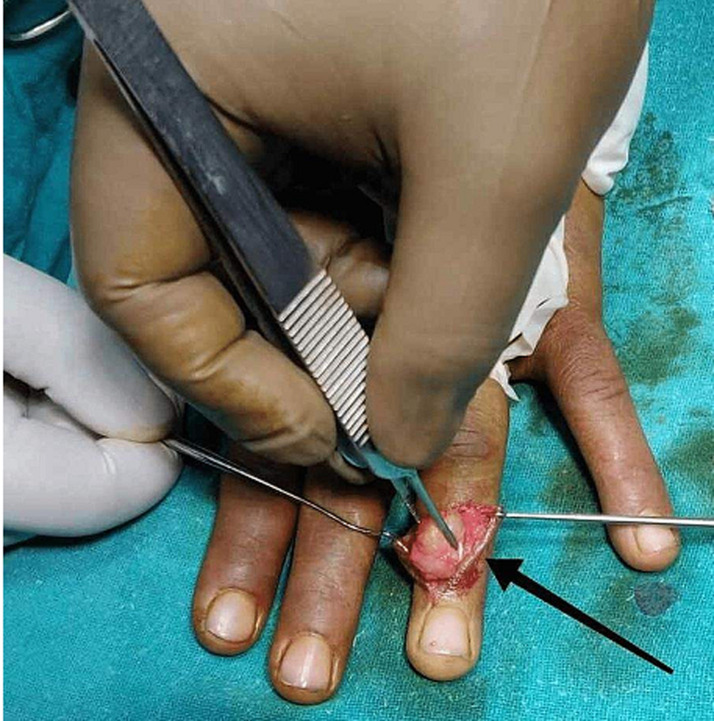
intraoperative image of removal of mass

**Figure 5:**
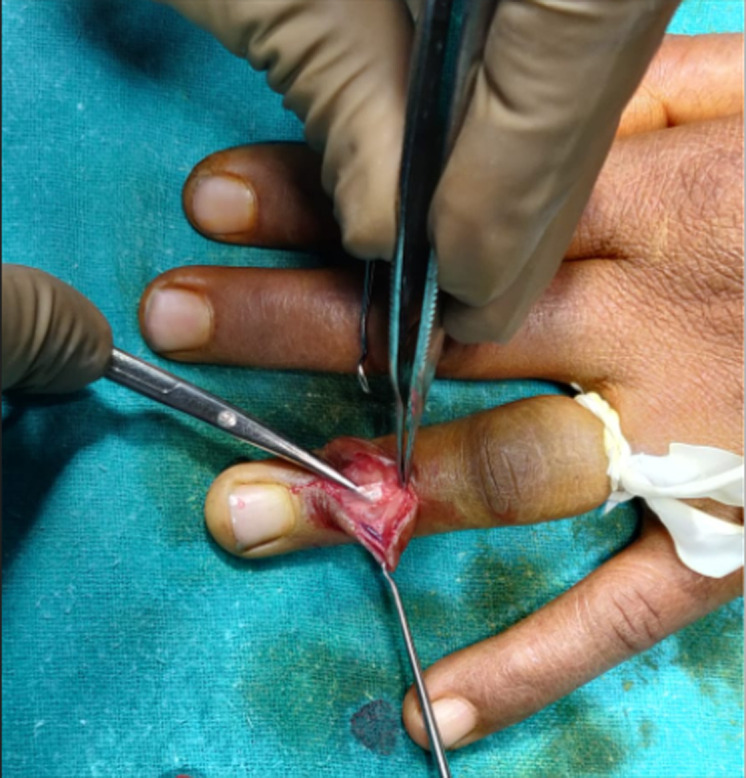
intraoperative image of after removal of mass, tendon was found to be intact

**Figure 6:**
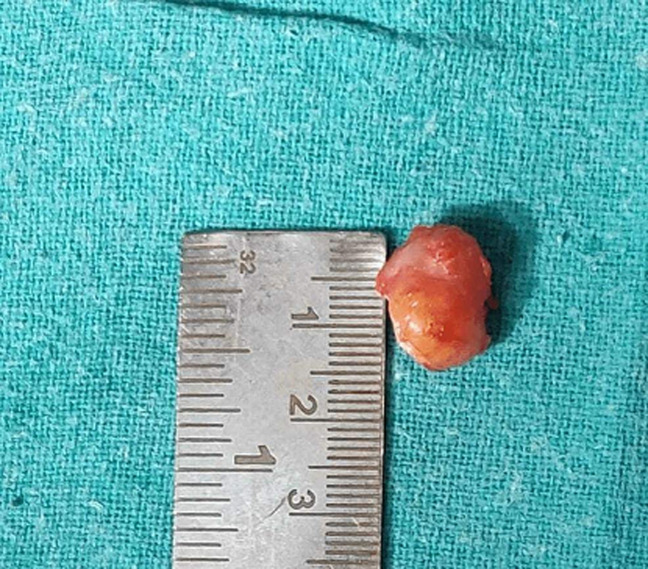
intraoperative image measuring size of tumour

**Therapeutic interventions:** in these types of rare GCT cases, surgical removal of mass is the definitive treatment. In our case, we gave a Z shaped incision (Figure 3) over the tumour, and it was found to be over the tendon sheath. The whole mass was removed in to-to (Figure 4, Figure 5, Figure 6) without damaging any tendon or neurovascular structure.

**Follow-up and outcome of interventions:** patient came for follow-up after 2 years and was assessed radiologically and clinically and was found to have no such evidence for recurrence of the tumour. On clinical examination, range of motion at interphalangeal joint and metacarpo phalayngeal joint of the deceased finger was within normal limits.

**Patient perspective:** the patient had a minimal hospital stay with fast recovery and no evidence of recurrence yet. The patient had a chief complaint of painful movements, which resolved with full range of movements. Also, follow-up visits and bed stay were minimal. Hence, the patient was more relieved, didn´t have to go through any bony deformity and was satisfied with his treatment.

**Informed consent:** informed written consent was obtained from the patient.

## Discussion

A giant cell tumour (GCT) is a neoplasm that develops from the connective tissue of the bone marrow's non-bone-forming unit. The distal end of the femur, the centre of the tibia, and the distal end of the radius are the most frequent sites, respectively [3]. Because the enormous cell resembled an osteoclast, it is sometimes called an osteoclastoma. Patients who are practically skeletally mature and between the ages of 15 and 40 are most frequently affected by this lesion in their third decade of life. As the bones are shallow, individuals with GCT of the hand appear extremely quickly. The most frequent presenting symptoms are pain and edema. Before a diagnosis is made, the majority of individuals have symptoms for an average of six months or less. The periosteum is seldom broken, even when cortical damage occurs. An all-lytic X-ray lesion is the defining feature of GCT. It can occasionally seem like a soap bubble, as in our instance, and is typically quirky. Typically, the lesion's edge that touches the cancellous bone is well-marked. Periosteal response can occur if bone morphology is altered. A significant finding of GCT fine-needle aspiration cytology (FNAC) is multi-nucleated large cells (osteoclast). Tumour's proliferative capacity is not brought by the normal osteoclastic giant cells. Multinucleated giant cells are created when growing mononuclear cells combine [5].

As in our case, it can occasionally appear like a soap bubble and is generally eccentric. Periosteal reaction can happen if the bone is damaged or surgically changed, despite the fact that it is uncommon. Multinucleated big cells are a common finding in bone GCT fine-needle aspiration cytology (FNAC) samples (osteoclast). A significant characteristic of the GCT is the surrounding adhesion of large cells to the clusters of mononuclear cells. The typical big osteoclastic giant cells of bone do not contribute to the tumour's ability for proliferation. Growing mononuclear cells merge to form multinucleated giant cells. The tendon sheath can rise to benign lesions called tenosynovial giant cell tumours. It is not apparent if these lesions are reactive masses or actual tumours. These lesions are frequently seen as isolated, single, subcutaneous soft tissue nodules on imaging, with weak T1 and T2 signals and mild enhancement. According to the most recent (2013) World Health Organization classification, tenosynovial giant cell tumour is the word used. Previously, they were referred to as localised or focal nodular synovitis, pigmented villonodular tumours of the tendon sheath, extra-articular pigmented villonodular tumours of the tendon sheath, and giant cell tumours of the tendon sheath (GCTTS). In 2013, these cancers were categorized by the World Health Organization (WHO). This categorization distinguishes between localised and diffuse tenosynovial giant cell tumours. Previously recognized giant cell tumours of the tendon sheath were included in the localised TGCT (GCTTS). Formerly known as nodular tenosynovitis diffuse TGCT includes both (PVNS) [2]. Chromosomal translocation occurs when a section or portion of a particular chromosome separates and is rearranged, causing the genetic material to move and altering the set of chromosomes. Certain areas on chromosomes 1 and 2 are involved in a translocation that occurs in these malignancies. Colony-stimulating factor-1, or CSF-1, is a kind of protein that is overproduced by cells with this translocation. The TGCT cells use CSF-1 to recruit the white blood cells to incorporate into the tumour and most likely cause the inflammatory changes caused by these tumours [6].

Internal fixation, prolonged curettage, intralesional curettage with adjuvant therapy, intralesional curettage with bone grafting, and curettage with curettage are among the therapeutic options available [7]. Traditional GCT in the remainder of the skeleton appears to show no lesion, but GCT of the hand does. An 18% frequency of foci are multicentric. The GCT in the hand is particularly aggressive despite not being a sarcoma and has a high risk of recurrence even after limited resection. Surgery usually leaves a serious bone deformity and a questionable reconstruction. Other treatment options include curettage and bone grafting, delayed curettage, resection with reconstruction, and even amputation [8].

## Conclusion

The significance of early diagnosis of GCT by FNAC cannot be understated, since management may prevent deformity and impairment through careful surgical planning prior to an excision biopsy. In certain situations, a combination of curettage, cryosurgery, and cementation seems to produce excellent outcomes. The recommended surgical procedure is local excision of the affected bone with autograft and/or allograft replacement. Recurrence rates for standard manual curettage are 40%, whereas they are 10- 20% with prolonged curettage. The hand's recurrent GCT is more aggressive and may need amputation, which deforms the hand's appearance and reduces its functionality. Our case report shows early presentation with early diagnosis, with early surgical intervention with postoperative vigorous physiotherapy can lead to regaining of normal anatomic and physiological functions of the affected joint.

## References

[1] Awad P (2021). Atypical Tenosynovial giant cell tumor of the foot and ankle: A case report. J Am Podiatr Med Assoc.

[2] Anderson WJ, Doyle LA (2021). Updates from the 2020 World Health Organization Classification of soft tissue and bone tumours. Histopathology.

[3] Allen EV (1929). Thromboangiitis obliterans: methods of diagnosis of chronic occlusive arterial lesions distal to the wrist with illustrative cases. Am J Med Sci.

[4] Enneking WF, Spanier SS, Goodman MA (1980). A system for the surgical staging of musculoskeletal sarcoma. Clin Orthop Relat Res.

[5] Gupta K, Dey P, Goldsmith R, Vasishta RK (2004). Comparison of cytologic features of giant-cell tumor and giant-cell tumor of tendon sheath. Diagn Cytopathol.

[6] Flevas DA, Karagiannis AA, Patsea ED, Kontogeorgakos VA, Chouliaras VT (2021). Arthroscopic removal of Tenosynovial giant-cell tumors of the cruciate ligaments. Presentation of two cases. J Orthop Case Rep.

[7] Cannarile MA, Weisser M, Jacob W, Jegg AM, Ries CH, Rüttinger D (2017). Colony-stimulating factor 1 receptor (CSF1R) inhibitors in cancer therapy. J Immunother Cancer.

[8] Jabir R, Makabori (2022). Januar & Zufar, Muhammad & Kurniawati, Tri. Giant Cell Tumor of the Middle Phalanx of Ring Finger Treated with Wide Excision and Reconstruction using Non-vascularized Fibular Graft: A Rare Case Report and Review of Literature.

